# Biological factors that impinge on Chagas disease drug development

**DOI:** 10.1017/S0031182017001469

**Published:** 2017-08-23

**Authors:** AMANDA F. FRANCISCO, SHIROMANI JAYAWARDHANA, MICHAEL D. LEWIS, MARTIN C. TAYLOR, JOHN M. KELLY

**Affiliations:** Department of Pathogen Molecular Biology, London School of Hygiene and Tropical Medicine, Keppel Street, London WC1E 7HT, UK

**Keywords:** Chagas disease, *Trypanosoma cruzi*, drug development, disease pathogenesis

## Abstract

Chagas disease is caused by infection with the insect-transmitted protozoan *Trypanosoma cruzi*, and is the most important parasitic infection in Latin America. The current drugs, benznidazole and nifurtimox, are characterized by limited efficacy and toxic side-effects, and treatment failures are frequently observed. The urgent need for new therapeutic approaches is being met by a combined effort from the academic and commercial sectors, together with major input from not-for-profit drug development consortia. With the disappointing outcomes of recent clinical trials against chronic Chagas disease, it has become clear that an incomplete understanding of parasite biology and disease pathogenesis is impacting negatively on the development of more effective drugs. In addition, technical issues, including difficulties in establishing parasitological cure in both human patients and animal models, have greatly complicated the assessment of drug efficacy. Here, we outline the major questions that need to be addressed and discuss technical innovations that can be exploited to accelerate the drug development pipeline.

## INTRODUCTION

Five to eight million people in Latin America are infected with the protozoan parasite *Trypanosoma cruzi*, the aetiologic agent of Chagas disease (Hashimoto and Yoshioka, 2012; Bern, 2015). Infections are spread primarily by blood-sucking triatomine bugs, although other means of transmission include the congenital route, contaminated food and drink, organ transplantation and blood transfusion. Chagas disease is also becoming a global public health problem, with significant numbers of symptomatic cases now being detected within migrant populations, particularly in the USA and Europe, where the estimates of those infected are 300 000 and 100 000, respectively (Bern *et al.* 2011; Pérez-Molina *et al.* 2012; Requena-Méndez *et al.* 2015).

Chagas disease has been divided into three discrete phases. The initial ‘acute’ stage, which occurs in the first 4–6 weeks post-infection, usually manifests as a mild and transient febrile condition, and in many cases, is asymptomatic. However, in children it can be more severe and sometimes fatal. With the development of a vigorous adaptive immune response, in which CD8^+^ IFN-*γ*^+^ T cells play a major role (Cardillo *et al.* 2015; Tarleton, 2015), the infection is suppressed, but sterile immunity is not achieved. This ‘indeterminate’ or ‘asymptomatic chronic’ stage is characterized by an intermittent and extremely low-level parasitaemia. However, ~30% of infected individuals eventually proceed to the ‘symptomatic chronic’ stage, often decades after the primary infection. Cardiomyopathy develops in the majority of these individuals, whilst a minority (approximately 10% of those infected) suffer digestive tract megasyndromes (Ribeiro *et al.* 2012; Cunha-Neto and Chevillard, 2014). Chagas disease is a major cause of premature death in many areas of South America.

The front-line drugs used to treat *T. cruzi* infections are the nitroheterocyclic compounds benznidazole and nifurtimox (Wilkinson and Kelly, 2009; Gaspar *et al.* 2015). Both have been in use for almost 50 years, despite widespread evidence of treatment failures (Molina *et al.* 2014; Morillo *et al.* 2015, 2017). Other drawbacks include the long treatment period (often 60 –90 days), the frequency and severity of side-effects, and the potential for cross-resistance, which arises from the requirement of these nitroheterocyclic agents to be activated by the same parasite mitochondrial nitroreductase, TcNTR-1 (Wilkinson *et al.* 2008; Mejia *et al.* 2012). Although benznidazole has proven to be effective at curing some acute and chronic *T. cruzi* infections, the extent to which it can prevent or alleviate chronic cardiac pathology remains uncertain (Molina-Berríos *et al.* 2013; Villar *et al.* 2014; Gruendling *et al.* 2015; Morillo *et al.* 2015). The only new compound recently advanced into clinical trials has been the anti-fungal agent posaconazole, which blocks ergosterol biosynthesis through inhibition of lanosterol 14α-demethylase (CYP51). Unfortunately, posaconazole proved to have limited efficacy against chronic infections (Molina *et al.* 2014; Francisco *et al.* 2015; Morillo *et al.* 2017), despite some initially promising outcomes in experimental animal models (Molina *et al.* 2000; Ferraz *et al.* 2007).

The urgent need to develop more effective therapy against Chagas disease is now being tackled by large international, multidisciplinary teams (Katsuno *et al.* 2015; Chatelain, 2016). These have introduced a more systematic framework to drug development by bringing together expertise from both the academic and commercial sectors. However, it is clear that progress is being limited by gaps in our knowledge of parasite biology and disease pathogenesis, and that further technical innovations are required to accelerate the pathway that stretches from lead compound optimization to pre-clinical testing. Below, we highlight these major biological questions and discuss various approaches that could help to streamline the drug development process.

## DOES PARASITE DIVERSITY IMPACT ON DRUG EFFICACY?

**Fig. 1.**
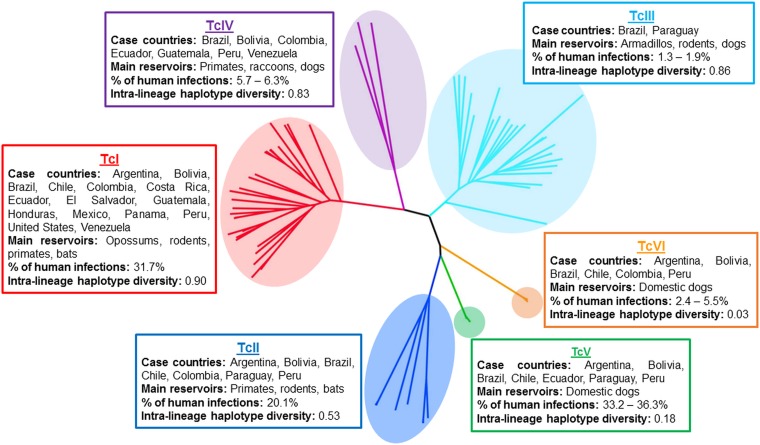
Key features of the *Trypanosoma cruzi* major genetic lineages. The phylogenetic tree was reconstructed using multi-locus microsatellite genotype data, adapted from Lewis *et al.* (2011). Haplotype diversity is based on mitochondrial gene sequences for COII and ND1 reported in Lewis *et al.* (2011), and the values shown indicate the probability that two randomly selected haplotypes will be different. The percentage of human infections is estimated from metadata compiled by Brenière *et al.* (2016), which encompass all isolates derived from human infections (*n* = 1902) and typed to each lineage. These values may reflect historical variation in sampling intensities between endemic areas.

*Trypanosoma cruzi* is a highly diverse species with genetic distances between major lineages greater than those between members of the *Trypanosoma brucei* species complex (Franzén *et al.* 2011). Our understanding of parasite taxonomy has been complicated further by evidence of genetic exchange and widespread detection of putative hybrid strains (Machado and Ayala, 2001; Brisse *et al.* 2003; Gaunt *et al.* 2003; Lewis *et al.* 2011). The geographical range of *T. cruzi* extends from southern Chile and Argentina, through Central America, into wide areas of the southern USA (Brenière *et al.* 2016). The parasite can be transmitted by more than 100 species of triatomine vector and is capable of infecting most, if not all, mammalian species that it encounters (Messenger *et al.* 2015). The taxonomic categorization of *T. cruzi* has been subject to long, and at times vigorous, debate. Currently, the species is divided into six discrete typing units (DTUs) designated TcI–TcVI (Zingales *et al.* 2012) (Fig. 1), although a seventh, Tcbat, has recently been proposed (Marcili *et al.* 2009; Pinto *et al.* 2012). TcI is the most geographically dispersed DTU, with a range stretching from the USA to Argentina, and although the other lineages are more localized, there is considerable overlap (for review, Miles *et al.* 2009; Brenière *et al.* 2016). This extensive diversity has prompted speculation as to whether there are correlations between parasite lineage, host preference, disease pathology and drug sensitivity.

All six DTUs are capable of infecting humans (Fig. 1); overall TcI and TcV infections are the most commonly identified, although other lineages predominate in some specific endogenous areas, such as TcII in parts of Brazil. Despite some circumstantial data, there has been no unequivocal evidence of a causative link between parasite diversity and disease outcome in humans. For example, suggestions that the absence of gastrointestinal megasyndromes in Venezuela compared with Brazil might be associated with genetic differences in the populations of circulating parasites have yet to be validated (Messenger *et al.* 2015). There is substantial intra-lineage genetic diversity, particularly within TcI, II, III and IV (Lewis *et al.* 2011); however, this has rarely been taken into account, because in most studies, genotyping is only conducted at the lineage level. Studies using experimental animal models do show that there can be important differences in virulence between individual strains (Schlemper *et al.* 1983; Postan *et al.* 1987; Espinoza *et al.* 2010; Rodriguez *et al.* 2014; Lewis *et al.* 2016), although no candidate genetic factors have been identified. The increasing availability of genomic technologies should enable progress to be made in delineating the extent to which parasite genetics contributes to disease outcome.

There have been multiple reports of wide divergence in the drug susceptibility of different *T. cruzi* strains. In a survey which encompassed representatives of DTUs I–VI, significant differences in benznidazole sensitivity were identified, but there was no correlation with parasite lineage (Villarreal *et al.* 2004). These results were consistent with data obtained from a panel of 28 parasite isolates from Colombia, where *in vitro* EC_50_ values against benznidazole ranged from 1 to 35 *µ*m (Mejia *et al.* 2012). Again, there was no obvious correlation between sensitivity and lineage (the panel contained DTU I and II strains), or with the biological origin of the parasites, either insect vector, small mammal or human. This extensive natural variation in benznidazole sensitivity was independent of *TcNTR-1* sequence, implying that it must be associated with additional factors. In another report, parasites belonging to each of the DTUs were tested *in vitro* against several nitroheterocylic drugs and other lead compounds (Moraes *et al.* 2014). Although parasite strains exhibited a range of susceptibilities to individual drugs (e.g. up to 8-fold in the case of nifurtimox), there was no evidence that any of the lineages were intrinsically more resistant to drugs in general. *In vivo* studies have been inconsistent in terms of linking drug susceptibility to parasite lineage. For example, while Toledo *et al.* (2003) found that TcI-infected mice were less frequently cured by benznidazole or itraconazole than TcII- or TcV-infected mice, they also observed extensive heterogeneity in drug sensitivity between strains within these lineages. Similar intra-lineage variations were also reported when the curative potential of benznidazole was assessed with a number of Brazilian strains (Teston *et al.* 2013). In a study with the other front line drug nifurtimox, no association was found between therapeutic effectiveness and parasite lineage (Oliveira *et al.* 2017).

In summary, although natural *T. cruzi* isolates can display large variations in drug susceptibility, there is little evidence to link this with their taxonomic designation at the DTU level. As a general observation, intra-lineage differences seem to be as extensive as those between lineages. This highlights that assessment of the ability of lead compounds to display *in vivo* activity against a wide panel of isolates, reflecting the diverse phylogeny and geographical range of *T. cruzi*, must be considered an integral step in the drug development pathway. The importance of this is emphasized further by the commonality of mixed infections (Bontempi *et al.* 2016).

## ARE ALL PARASITE LIFE-CYCLE STAGES EQUALLY SUSCEPTIBLE TO CHEMOTHERAPY?

From the drug development perspective, there are a number of important questions relating to the *T. cruzi* life-cycle that need to be addressed: (i) Is it necessary to kill all developmental forms to produce a curative outcome? (ii) Are all developmental forms equally susceptible to trypanocidal compounds? (iii) Is there a point in the life-cycle during chronic stage infections where the parasites enter a biochemically quiescent or dormant phase? The *T. cruzi* life-cycle involves a series of differentiation steps, in which the parasite passes through both replicative and non-replicative stages. In the classical text-book version of the mammalian life-cycle, which has been established for more than a century, insect-transmitted non-replicating metacyclic trypomastigotes invade host cells, differentiate into small round-shaped, non-flagellated intracellular amastigotes, and then divide by binary fission in the cytosol. When they reach a threshold level, which may be several hundred per infected cell, they differentiate into non-dividing flagellated trypomastigotes, which are released following lysis of the host cell. The trypomastigotes can then re-invade other cells, or be taken up in a bloodmeal by a feeding triatomine bug.

**Fig. 2.**
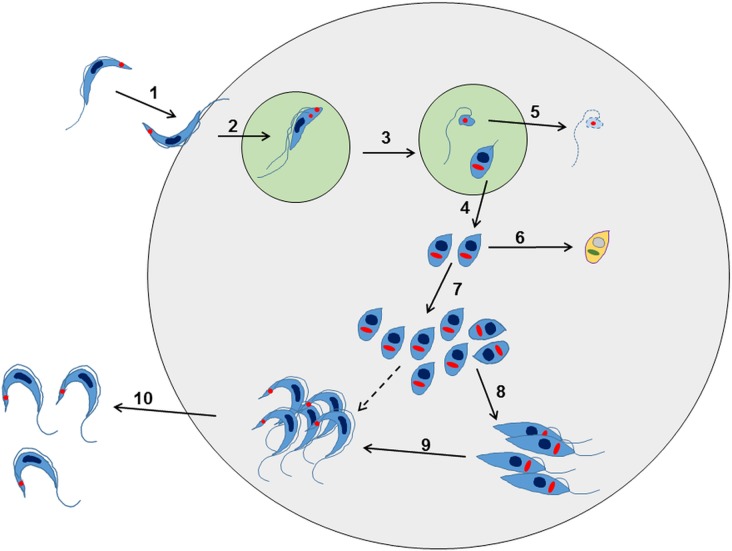
Overview of the intracellular life cycle of *Trypanosoma cruzi* in the mammalian host. (1) The metacyclic trypomastigote binds to receptors on the host cell surface resulting in the parasite being taken up into a parasitophorous vacuole. This occurs regardless of whether or not the host cell is phagocytic. (2) The parasite undergoes an asymmetric cell division following replication of the kinetoplast (red circle) and flagellum, but not the nucleus (Kurup and Tarleton, 2014). (3) This results in one daughter cell being a replication competent amastigote with a short flagellum, and the other being a dysnuclear flagellated cytoplasmic fragment. (4) The amastigote escapes into the cytoplasm and begins replication by binary fission. (5) The remaining parasite component is degraded by the proteasome and its antigens are presented on the surface. (6) Some amastigotes may become metabolically quiescent, although this is yet to be proven. Such amastigotes could reside long term in chronically infected tissue. (7) The amastigotes continue to replicate. (8) Amastigotes differentiate into an intracellular epimastigote-like form. It is not clear whether this is an obligate stage, or if they can go straight from amastigotes to trypomastigotes (dashed arrow). (9) The parasites finally differentiate into the flagellated bloodstream trypomastigotes, lyse the host cell and escape into the bloodstream or tissue fluids (10).

Further research has revealed that this established view of the life-cycle is rather superficial and that in reality, the process is almost certainly more complex (Fig. 2). For example, evidence for an intracellular epimastigote-like stage has been intermittently reported (for review, Tyler and Engman, 2001), although it is unclear whether this enigmatic form is simply an intermediate in the amastigote to trypomastigote transition, or represents an obligate intracellular stage of the life-cycle, with a distinct role *in vivo*. Similarly, amastigote-like forms with short flagella, termed sphaeromastigotes, have also been widely reported, although these probably represent intermediate forms in the transition to epimastigotes, rather than distinct life-cycle stages (Tyler and Engman, 2001). Recently, trypomastigotes have also been shown to have the capacity to differentiate into an epimastigote-like morphological form, after transition through an amastigote-like intermediate. These recently differentiated epimastigotes (rdEpi) display a distinct proteomic fingerprint, are complement-resistant, able to invade phagocytic and cardiac cells (but not fibroblasts or epithelial cells), and can initiate an infection in mice (Kessler *et al.* 2017). It has also been demonstrated that the initial differentiation from the metacyclic trypomastigote involves an asymmetric cell division (Fig. 2), which results in one amastigote and one ‘zoid’ – a cell with a kinetoplast, but no nucleus. The zoid quickly dies and is degraded by the host cell machinery with some of its antigens being presented on the infected cell surface (Kurup and Tarleton, 2014). The role(s) of the intermediate *in vivo* forms are not well defined, and it is unclear whether their sensitivity (or otherwise) to test compounds is adequately captured using current *in vitro* screening systems. Efficacy testing against trypomastigotes is now routinely incorporated into drug screening protocols (Cortes *et al.* 2015; Guedes-da-Silva *et al.* 2016). However, the sensitivity of other intracellular and/or intermediate stages is intrinsically difficult to establish, and it is unknown whether it is necessary to target each of these morphological forms to eradicate an infection.

The importance of understanding the interplay between drug activity and the parasite life-cycle has been highlighted by studies with CYP51 inhibitors, such as posaconazole and ravuconazole. Although these drugs have low nanomolar EC_50_ values against a range of *T. cruzi* strains, they seem unable to completely clear parasites from infected mammalian cells in culture (Moraes *et al.* 2014). Consistent with this, studies in murine models have shown that although posaconazole is highly effective at reducing the parasite burden when administered at 10 mg kg^−1^ day^−1^ for 25 days, it does not eliminate the infection, even when the dose is increased 10-fold, or when the treatment period extended to 40 days (Khare *et al.* 2015). One possibility is that treatment could induce resistance by promoting higher level expression of the lanosterol 14α-demethylase target. Alternatively, there could be a sub-population of dormant, metabolically quiescent parasites within infected host cells, which have a reduced requirement for ergosterol biosynthesis. Addressing if this is the case, must be considered a major research goal, particularly because the existence of such parasites might have broader relevance for many other classes of drug.

**Fig. 3.**
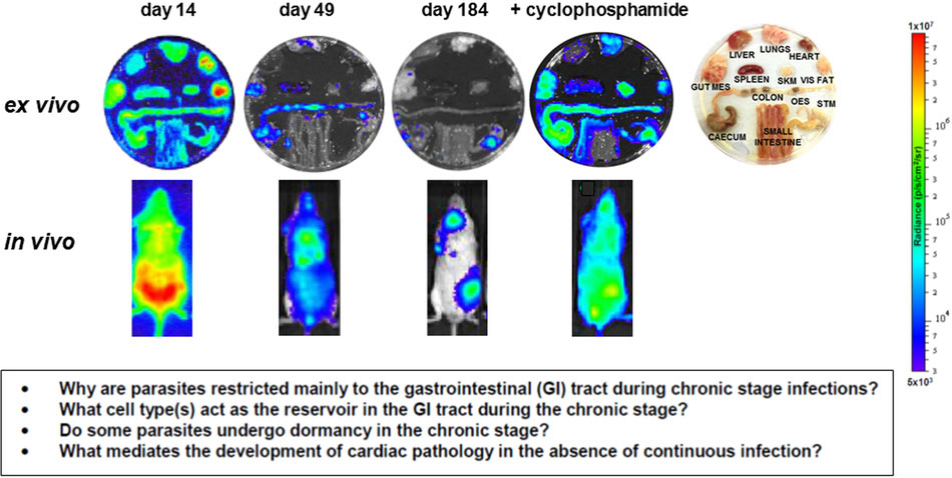
Parasite tropism during *Trypanosoma cruzi* infections in a mouse model. BALB/c mice infected with bioluminescent *T. cruzi* strain CL Brener (Lewis *et al.* 2014) were imaged at various stages post-infection, as indicated. Upper; *ex vivo* imaging of organs/tissues removed from mice and soaked in D-luciferin. The identity of each organ/tissue is indicated (right). Lower; *in vivo* imaging of infected mice. The bioluminescence images on the right-hand side are of a chronically infected mouse which has been immunosuppressed by cyclophosphamide treatment (Lewis *et al.* 2014). Heat-maps are on log10 scales and indicate intensity of bioluminescence from low (blue) to high (red). Inset: summary of the major unanswered questions.

In addition to the above, it could be that metabolically dormant parasites exist in some tissue niches during chronic infections. Investigating this is a major technical challenge, since parasites are present in extremely low numbers during the chronic stage. As outlined below, bioluminescence imaging allows parasites to be localized to specific organs and tissues in murine models, with the colon and/or stomach identified as the major reservoirs during the chronic stage (Fig. 3) (Lewis *et al.* 2014,2016). However, the technology is not sufficiently sensitive or applicable to allow the microscopic detection of individual infected cells. One strategy currently being developed involves the generation of parasites that express dual bioluminescence:fluorescence reporters, so that infected foci in tissues can be pinpointed, excised and sectioned, and then intracellular parasites visualized by fluorescence microscopy (Taylor *et al.* unpublished). This type of approach will be essential if the phenotype of the reservoir host cells is to be determined, the metabolic and replicative status of the residual parasites defined, and their response to trypanocidal drugs assessed.

## DOES PARASITE TROPISM DURING CHRONIC INFECTIONS HAVE THERAPEUTIC IMPLICATIONS?

In humans, infections with *T. cruzi* are considered to be life-long (Álvarez *et al.* 2014; Cardoso *et al.* 2016). However, during the chronic phase, parasites are highly focal, present at extremely low levels, and only sporadically detectable in the bloodstream. This can limit the accurate diagnosis of on-going infections, even with PCR-based methodology (Schijman *et al.* 2011), and is an important complicating factor in clinical trials. By necessity, most studies on parasite tropism and persistence during human chronic infections have focused on tissue samples retrieved at autopsy, or following organ transplantation. The degree to which these findings are relevant to the majority of infected people is uncertain, and as a result, parasite tropism in asymptomatic individuals is poorly understood.

Intracellular amastigotes have been detected in some chagasic heart samples using histology (Benvenuti *et al.* 2014; Kransdorf *et al.* 2016), and more frequently using PCR or antibody-based techniques (Bellotti *et al.* 1996; Schijman *et al.* 2004; Burgos *et al.* 2010). Evidence from transplantation-linked transmission, in both endemic and non-endemic regions (Kun *et al.* 2009; Huprikar *et al.* 2013), suggests that parasites can be present in a number of organs, with infections more common after heart transplants, than those involving kidney or liver. Parasite DNA has also been detected in the oesophagus (Vago *et al.* 1996; Lages-Silva *et al.* 2001) and adipose tissue (Ferreira *et al.* 2011) during chronic infections, and parasites can become widely disseminated following reactivation of Chagas disease in immunosuppressed patients (see Fig. 3, as an example in a murine model), or those co-infected with HIV (for review, Lattes and Lasala, 2014). CNS involvement leading to meningoencephalitis is a common outcome in these situations (Cordova *et al.* 2008; Diazgranados, *et al.* 2009; Yasukawa *et al.* 2014).

Current knowledge on infection dynamics and parasite tropism during chronic *T. cruzi* infections in humans is insufficient to identify which tissue sites are important in terms of drug targeting and bioavailablity, or to determine if specific organs or tissues have a role in recrudescence. Given the practical difficulties in addressing these questions in infected patients, predictive experimental models have been at the forefront of the research effort. Animal models include dogs (Santos *et al.* 2016), primates (Vitelli-Avelar *et al.* 2017), chickens (Teixeira *et al.* 2011) and most commonly, mice, where several model systems are available which mimic aspects of disease pathology in humans (Eickhoff *et al.* 2010; Olivieri *et al.* 2010; Molina-Berríos *et al.* 2013; Sbaraglini *et al.* 2016). However, even in experimental models, difficulties in monitoring parasite burden and location during chronic infections have been a limiting step. With mice, these issues have been partially resolved by the development of highly sensitive bioluminescence imaging procedures, which for the first time enable chronic infections to be assessed in real time (Lewis *et al.* 2014, 2015). The system, which utilizes genetically modified parasites that express red-shifted luciferase (Branchini *et al.* 2010), has a limit of detection close to 100 parasites in inoculated mice and allows infections to be monitored in individual animals for more than a year (Lewis *et al.* 2014). In the BALB/c mouse -*T. cruzi* CL Brener (DTU VI) model, the infection peaks on day 14 post-inoculation (Fig. 3). Following induction of an adaptive immune response, the parasite burden then decreases by two to three orders of magnitude over the next 30–40 days, as the infection progresses to the chronic stage. Long-term infections are characterized by a dynamic profile, in which bioluminescence foci appear and disappear over a period of hours, in an apparently stochastic manner (Lewis *et al.* 2014). Similar patterns of infection occur with other parasite lineages and mouse strains, although with some differences in the precise timing of progression, presumably reflecting the influence of host and parasite genetics (Taylor *et al.* 2015; Lewis *et al.* 2016).

In the murine acute stage, the infection is pan-tropic, with parasites easily detectable by *ex vivo* imaging in all organs and tissues (Fig. 3). However, in the chronic stage, the colon and/or stomach are the primary sites of infection. Other organs, including the heart, are infected only sporadically, with the extent of this varying in different host:parasite strain combinations (Lewis *et al.* 2016). Myocarditis and heart fibrosis can develop in the absence of end-point cardiac infection, implying that the continuous presence of the parasite in the heart is not a pre-requisite for chagasic pathology. These data suggest a model where the gut is a permissive immunological niche that tolerates continuous low-level infection, with periodic trafficking of parasites, or more likely, parasite-infected cells, from this reservoir to other sites, including the heart. This leads to the generation of intermittent inflammatory immune responses that eliminate the transient infections in non-gut sites, but can result in collateral damage to surrounding tissue (Lewis and Kelly, 2016). It is implicit in this model that drug-mediated elimination of parasites from the gut reservoir sites should lead to parasitological cure in asymptomatic, immunocompetent individuals, with the immune system eradicating the remaining parasites from non-gut sites. While this model has yet to be proven, it does generate a number of questions with implications for drug development. First, what is the nature of the host cells in which parasites persist within the gut reservoir sites, and what is their immunological and metabolic status? Second, do parasites in these sites display dormancy, and if so, does this affect their sensitivity to therapeutic treatment? Third, do the transient bioluminescent foci in chronically infected mice represent infected cells undergoing trafficking, and what is their fate in an immunocompetent individual? Finally, can these insights into parasite tropism and persistence be extended from murine models to human patients?

The current inability to reliably cure chronic *T. cruzi* infections has led to speculation that the parasite might be able to survive in organs/tissues where drug access is limited. However, this appears not to be the case with nitroheterocyclic drugs, at least in mice. Treatment failures, in both acute and chronic infections, are not linked to a single or predominant site of recrudescence (Francisco *et al.* 2015, 2016). Consistent with this, a detailed study of benznidazole pharmacokinetics has revealed that inadequate bio-distribution is unlikely to be responsible for therapeutic failure during chronic phase murine infections (Perin *et al.* 2017). With posaconazole, the situation is less clear-cut. Although adipose tissue has been identified as a frequent site of cyclophosphamide-induced relapse after non-curative treatment of acute stage infections (Francisco *et al.* 2015), this was not observed in all mice. Adipose tissue has been implicated as a possible reservoir of recrudescence in other parasitic infections, including African trypanosomiasis (Trindade *et al.* 2016; Tanowitz *et al.* 2017); however, further work will be required before definitive conclusions can be drawn about the situation in *T. cruzi*.

## DOES DRUG TREATMENT PREVENT OR ALLEVIATE CHRONIC DISEASE PATHOLOGY?

At a population level, the major health and economic burdens associated with Chagas disease result from chronic cardiac pathology. However, chronic stage studies, particularly on drug efficacy and disease pathogenesis, are made difficult by the scarce and highly focal nature of *T. cruzi* infection. As a result, the major research effort has focussed on the acute stage, where the monitoring of parasite load and the assessment of tissue tropism are more straightforward. There has been much discussion within the community on the underlying causes of chronic Chagas disease pathology (Gironès *et al.* 2005; Kierszenbaum, 2005; Gutierrez *et al.* 2009; Bonney and Engman, 2015). The context for this debate has been the inability to routinely detect parasites in the hearts of patients with cardiac damage and the non-specific (and sometimes autoreactive) polyclonal B-cell and T-cell responses that are characteristic of *T. cruzi* infection (Iwai *et al.* 2005; Bermejo *et al.* 2011). The central question has been: does chronic stage cardiac pathology develop as a consequence of autoimmunity, or does it result from parasite persistence and the generation of aberrant inflammatory responses within target organs? There is now a strong consensus that the presence of the parasite is a pre-requisite for heart pathology (for review, Bonney and Engman, 2015), although as mentioned above, the mode of cardiac infection may be one of episodic re-invasion rather than continuous persistence (Lewis and Kelly, 2016).

Cardiomyopathy develops in 20–30% of *T. cruzi-*infected patients. Progressive heart failure, thromboembolism, ventricular arrhythmia, stroke and sudden death are common outcomes (Rossi *et al.* 2003; Carod-Artal, 2010; Carod-Artal and Gascon, 2010). Patient management is based on standard protocols for treating progressive cardiac failure (Ribeiro *et al.* 2012), despite the lack of robust randomized clinical trials to validate their use in cases of chagasic heart disease. The ability of drug treatment to prevent or alleviate the development of cardiac pathology is unresolved, and controversial. Despite this, the consensus view is that treatment should be offered to patients infected with *T. cruzi*, irrespective of their disease status. The validity of this approach is one of the central debates in the Chagas disease field. There is reasonable evidence from experimental models that curative treatment of acute stage infections results in reduced disease pathology in the longer term (Davies *et al.* 2010; Molina-Berríos *et al.* 2013; Assíria Fontes Martins *et al.* 2015; Gruendling *et al.* 2015). With chronic stage infections, the data are less clear-cut (Villar *et al.* 2014). For example, in a recent large multi-centre, randomized clinical trial, no significant improvements in terms of cardiomyopathy were observed 5 years after benznidazole treatment (BENEFIT trial; Morillo *et al.* 2015). However, because evidence of cardiomyopathy was a pre-requisite for enrolment in this trial, it has not been possible to draw conclusions about the type of outcomes that might be achievable by treating asymptomatic individuals.

Clinical trials to assess the link between anti-parasitic treatment and reductions in pathology present numerous challenges. These include the long-term and diverse nature of the human disease, the toxicity of current drugs, the resulting compliance issues, and difficulties in demonstrating parasitological cure. This is complicated further by other variables such as the severity or otherwise of the acute stage infection, the possibility of re-infection/co-infection, host and parasite genetics, environmental factors and immune status. With advances in imaging technology, which allow real-time monitoring of chronic stage infections, it should be feasible to better exploit predictive animal models to investigate the rationale for using anti-parasitic drugs to prevent or alleviate symptomatic Chagas disease. Data from such experiments will be invaluable for informing the design of clinical trials aimed at establishing, for example, if there is a post-infection time limit within which curative therapy has to be administered to significantly impact on the development of cardiac pathology. The outcome of such studies should have major implications for the 5–8 million people infected with *T. cruzi.* However, it should be noted that currently, only 1% of those infected have access to diagnosis and treatment (DNDi, 2015).

### Concluding remarks

The development of more effective drugs against Chagas disease is a major challenge for the biomedical research community. The complexity of the infection, combined with our limited understanding of parasite biology and disease pathogenesis are major factors that inhibit progress in this area. Addressing these problems will require a twin track strategy; basic research to address the questions outlined in this review, and applied research, with input from the not-for-profit consortia and the commercial sector, to exploit the resulting opportunities and fast-forward the drug development pipeline.
